# No long-term effect of a 2-days intervention on how to prepare homemade food, on toddlers’ skepticism for new food and intake of fruits and vegetables and sweet beverages: a randomized, controlled trial

**DOI:** 10.1186/s13104-017-2931-z

**Published:** 2017-11-21

**Authors:** C. Beinert, S. Hernes, M. Haugen, N. C. Øverby

**Affiliations:** 1 Department of Public Health, Sport and Nutrition, University of Agder, PO 422, 4604 Kristiansand, Norway; 2 Department of Environmental Exposure and Epidemiology, Norwegian Institute of Public Health, Nydalen, P.Box 4404, 0403 Oslo, Norway; 3 Department of Public Health, Sport and Nutrition, Faculty of Health and Sport Sciences, University of Agder, PO Box 422, 4604 Kristiansand, Norway

**Keywords:** Food intake, Cooking course, Toddlers, Food skepticism

## Abstract

**Objective:**

Optimal nutrition from early age reduces the risk of developing non-communicable diseases later in life. The aim of this study was to examine the long-term effect on toddlers’ fruit and vegetable intake and sweet beverages, and skepticism for new food, of a 2-days’ intervention on how to prepare homemade food for toddlers.

**Results:**

The effect of the cooking intervention was evaluated by a randomized, controlled trial where 110 parents of 4–6 months old infants were included. Child diet and food skepticism were measured at 6, 15 and 24 months of age. There were no differences between the control and intervention group in the consumption of fruits and vegetables and intake of water or sweet beverages at 15 and 24 months. There were no differences between the control and intervention group, respectively, in percentage reporting having children who were skeptical regarding new food at baseline (29% vs 20%, p = .372), nor at 3 and 9 months after the intervention (20 vs 18%, p = .804 and 43% vs 32%, p = .383). The intervention did not influence intake of fruits and vegetables, nor did it reduce food skepticism among toddlers.

*Trial registration* first food for infants ISRCTN45864056, 20.05.2016. Retrospectively registered

**Electronic supplementary material:**

The online version of this article (10.1186/s13104-017-2931-z) contains supplementary material, which is available to authorized users.

## Introduction

The World Health Organization (WHO) states that in the first 2 years of a child’s life, optimal nutrition fosters growth and improves cognitive development [1]. They further acknowledge that optimal nutrition reduces the risk of becoming overweight or obese and developing non-communicable diseases (NCDs) later in life [2, 3]. This highlights the importance of child nutrition from early age.

The Norwegian Directorate of Health’s dietary guidelines for infants and toddlers [4] recommend exclusive breastfeeding the first 6 months of life, and introducing a variety of solid foods from 6 months with a special focus on fruits and vegetables, fish, and whole grain. In this study, we address two known barriers to a varied diet in children. One barrier to a varied diet is food neophobia, or skepticism to new food, which have shown to yield lower intakes of fruits and vegetables as well as fish [5]. Such skepticism peaks naturally at the age of two [6] and interventions regarding how to reduce levels of food skepticism before this age have been called for [5].

Further, there has been a decrease in the time spent on cooking and home preparation of family meals over the last few decades [7, 8] and studies have showed that being busy with work inside and outside the home, is regarded as a barrier for preparing “healthy” food by parents [9]. Virudachalam et al. [10] found that parents’ intervention preferences regarding food preparation for children, where to learn how to cook their usual meals in a healthier way and to participate in professionally taught classes.

In 2012, we conducted an intervention among parents with infants aiming to improve diet, food skepticism, weight and vitamin D and cholesterol status. We have previously reported that the intervention had a positive effect on High Density Lipoprotein (HDL)-cholesterol at age 24 months [11], however no effect on weight development and vitamin D status. We particularly presented dietary differences as lower prevalence of commercially produced dinners and porridges, however we have not presented results on details of fruit and vegetable intake, beverages and food skepticism. With this background, the aim of this paper was to examine the long-term effect on food skepticism and intake of fruit and vegetables and beverages, of a 2-days cooking course on how to prepare homemade food for toddlers conducted thru a RCT.

## Main text

### Method

#### Study design and subjects

This randomized controlled trial (RCT) was performed in 2012–2015 in a city area in Norway (Kristiansand). In total 110 parents of 4–6-month old infants were recruited through health care centers. Informed written consent was obtained from the parents/guardians of each child participating in this study. Of the 110 parents, 56 were randomly allocated to the intervention group where the parents took part in a 2-day cooking class with theoretical and practical lessons, and 54 were allocated to the control group which did not receive anything other than usual care. Parents completed a questionnaire when the child was 6, 15 and 24 months old. Characteristics of the included participants are presented in Table 1. There were no significant differences regarding demographic variables between the two groups at either time points. The participants were highly educated, few were smokers and most were married or cohabitants (Table 1). Follow-up evaluations were performed when the children were 15 and 24 months old. At 15 and 24 months of age, blood was drawn from the child’s index finger and results have been presented [11]. At the end of the study two randomly selected participants received 5000 NOK. Information about this was given during recruitment as a positive aspect of participating, acknowledging that participating in research is time consuming and for motivating for participation.

**Table 1:** Background information of the participants at baseline, 15 and 24 months of age

	Baseline: intervention (n = 53) n (%)	Baseline: control (n = 51) n (%)	p value^1^ (of means)	15 month: intervention (n = 44) n (%)	15 month: control (n = 27) n (%)	p value^1^ (of means)	24 month: intervention (n = 40) n (%)	24 month: control (n = 24) n (%)	p value^1^ (of means)
Age: (mean)									
Mother	30.3 (4.6)	30.9 (4.4)	.500	31.5 (4.8)	31.6 (3.1)	.700	32.2 (4.7)	32.5 (3.7)	.718
Father	32.0 (5.1)	33.7 (5.4)	.111	33.4 (6.2)	33.1 (3.8)	.741	33.9 (5.2)	33.7 (3.4)	.848
Educational level:									
Mother			.096			.678			.845
No college/university degree	6 (11)	10 (20)		4 (10)	2 (7)		5 (12.5)	2 (8)	
College/university ≤ 4 years	22 (42)	25 (49)		19 (43)	14 (52)		16 (40)	12 (50)	
College/university ≥ 4 years	25 (47)	16 (31)		21 (48)	11 (41)		19 (47.5)	10 (42)	
Father			.200			.464			.960
No college/university degree	15 (28)	20 (39)		16 (36)	10 (37)		13 (32.5)	7 (29)	
College/university ≤ 4 years or less	13 (25)	11 (22)		9 (20)	7 (26)		11 (27.5)	6 (25)	
College/university ≥ 4 years	23 (43)	19 (37)		19 (43)	10 (37)		16 (40)	11 (46)	
Smoking:									
Mother			.103			.409			.733
Daily	0 (0)	1 (2)		0 (0)	1 (4)		0 (0)	0 (0)	
Sometimes	0 (0)	2 (4)		3 (7)	2 (7)		1 (2.5)	1 (4)	
Never	53 (100)	48 (94)		41 (93)	24 (89)		39 (97.5)	23 (96)	
Father			.175			.695			.724
Daily	2 (4)	5 (10)		0 (0)	1 (4)		1 (2.5)	1 (4)	
Sometimes	1 (2)	2 (4)		3 (7)	2 (7)		3 (7.5)	2 (8)	
Never	50 (94)	44 (86)		41 (93)	24 (89)		36 (90)	21 (87.5)	
Mothers family situation:			.207			.148			.076
Married	32 (60)	24 (47)		31 (70)	14 (52)		31 (77.5)	14 (58)	
Cohabitant	20 (38)	26 (51)		12 (27)	12 (44)		9 (22.5)	8 (33)	
Single parent	1 (2)	1 (2)		1 (2)	1 (4)		0 (0)	1 (4)	

^1^Chi square test

#### Intervention course details

The intervention courses started in November 2012 and were given to five groups. Each of the two course days lasted 4 h, and parents were given theoretical information about an infant’s first food as well as cooking practice for making nutritious and varied dishes. Details of the interventions are given elsewhere [11]. In short, the first day focused on the introduction of the first solid food for infants. The theory referred to regular meals, water as a thirst quencher, iron-rich food, nutritious fruit purées, porridges, bread and toppings. The participants were informed about the importance of letting infants taste as many new food items as possible before the age of 2 years to prevent food neophobia and food skepticism. The participants prepared various kinds of fruit purées, porridges, breads, and nutritious toppings in the cooking classes. The second day, nutritious dinner meals were demonstrated with purées of carrots, potatoes, broccoli, sweet potatoes cauliflower, avocado and rutabaga. Purées of vegetables with tomatoes and cheese, chicken and tuna dishes with peas purée were also introduced [11].

#### Food consumption

Child food consumption was assessed with food frequency questionnaires at ages 6 (see Additional file 1), 15 and 24 months. All participants filled in questionnaires at baseline (6 months of age), and at 15 and 24 months of age. How often the child consumed different fruits and vegetables, water, sweet beverages were asked for. The response options ranged from “never/seldom” to “four times per day or more”. Responses were recoded to times per day. In Table 2 total consumption of fruits (sum of several fruits as apples, bananas, pears etc.) and vegetables are presented. The sum of sweet beverage includes lemonade and soft drinks with sugar and sweetened versions, fruit juices and nectars. We included both juices and soft drinks in this category as they are beverages with energy and few nutrients. Skepticism regarding new food was measured with only one question; “to which degree do you feel that your child is skeptical when new food is introduced”. Response alternatives ranged from 1 to 6 where 1 was not skeptic and 6 was very skeptical. We dichotomized the responses; 1–3 not skeptical, and 4–6 skeptical.

**Table 2:** Consumption of food and drinks (times/day) at baseline, at 15 and 24 months of age, for intervention and control group [mean (SD) and median Q1, Q3]

	Baseline					15 months of age					24 months of age				
	Intervention		Control		p value^1^	Intervention		Control		p value^1^	Intervention		Control		p value^1^
	(n = 53) Mean (SD)	Median (Q1, Q3)	(n = 51) Mean (SD)	Median (Q1, Q3)		(n = 44) Mean (SD)	Median (Q1, Q3)	(n = 27) Mean (SD)	Median (Q1, Q3)		(n = 40) Mean (SD)	Median (Q1, Q3)	(n = 24) Mean (SD)	Median Q1, Q3	
Water	1.7 (1.94)	1 (.0, 3)	1.39 (2.14)	.7 (.0, 2)	.138	5.4 (2.08)	7 (4.0, 7.0)	5.69 (1.91)	7 (4, 7)	.581	5.35 (2.1)	7 (3, 7)	5.8 (1.7)	7 (4, 7)	.378
Sweet beverages^2^	.27 (.15)	.0 (.0)	.14 (.66)	.0 (.0, .0)	.361	.59 (.0, .7)	.2 (.0, .78)	.56 (.66)	.28 (.14, .77)	.446	1.05 (.87)	.84 (.28, 1.56)	.89 (.64)	.99 (.35, 1.28)	.527
Commercially made porridge	1.27 (.89)	1 (.28, 2)	1.49 (.99)	1.35 (1, 2)	.314	.42 (.69)	.0 (.0, .56)	1.05 (1.51)	.71 (.0, 1.8)	.022	.04 (.12)	.0 (.0, .0)	.01 (.06)	.0 (.0, .0)	.201
Homemade porridge	.21 (.54)	.0 (.0, .0)	.25 (.56)	.0 (.0, .0)	.782	.56 (.69)	.28 (.0, 1)	.14 (.27)	.0 (.0, .28)	.008	.33 (.44)	.28 (.0, .49)	.24 (.35)	.0 (.0, .49)	.232
Fruit and berries^3^	1.02 (1.16)	.7 (.56, 1)	.93 (.67)	1 (.28, 1.28)	.597	1.66 (1.14)	1.4 (.99, 2.09)	1.7 (.76)	1.7 (1.1, 2.4)	.262	2.2 (1.3)	2 (1.36, 2.9)	2.69 (1.67)	2.4 (1.5, 3.27)	.107
Vegetables^4^	–	–	–	–	–	1.79 (1.06)	1.5 (.84, 2.54)	2.2 (1.54)	1.6 (1.1, 2.9)	.150	1.95 (1.37)	1.7 (.84, 2.97)	1.93 (1)	1.7 (1.7, 2.69)	.593

^1^Mann-Whithney U-test

^2^Includes lemonades with sugar and sweetened, sodas with sugar and sweetened, fruit juices and nectars

^3^Includes total amount of fruits and berries, both fresh and industrialized produced purees

^4^Consumption of total amount of vegetables. Not calculated for baseline since most of the vegetables were included in industrialized produced dinners, with different % of vegetables

#### Analysis

IBM SPSS Statistics 24 was used to analyze the data. Dietary data are presented as both means with standard deviations and median with interquartile range. As the dietary data was skewed, the non-parametric test, Mann-U-Whitney was used to analyze differences in food intake at baseline, 15 and 24 months between intervention and control group. Chi square was used to analyze differences in food skepticism between the two groups.

### Results

At age 24 months the intervention group drank water 5.35 (2.1) (mean SD) times/day vs 5.8 (1.7) times in the control group. The intervention group drank sweet beverages 1.05 (.87) times/day vs .89 (.64) times per day in the control group, analyzed with Mann–Whitney U-test. Mean vegetable consumption was approximately 1.95 (1.37 (intervention) and 1 (control)) times per day in both groups. None of these differences were significant. There were no differences in the consumption of food and drinks between the control and intervention group at 15 months either (Table 2), except for commercially made porridge [.42 (.69) vs 1.05 (1.51), p = .058] and homemade porridge [.56 (.69) vs .14 (.27), p = .002] at 15 month follow-up, as reported in a previous publication [11]. There were no differences between the control and intervention group (Table 3), respectively, in percentage reporting having children who were skeptical regarding new food (29% vs. 20%, p = .372). There were still no differences in percentage being skeptical at 15 and 24 months after the intervention between control and intervention group respectively (20 vs 18%, p = .804 and 43% vs 32%, p = .383).

**Table 3:** Number (%) being skeptic to new food and number (%) of parents continuing to expose child to new food even if they do not want to taste

	Baseline			15 months			24 months		
	Intervention N = 46 n (%)	Control (n = 28) n (%)	p value^a^	Intervention N = 51 n (%)	Control N = 25 n (%)	p value	Intervention n (%)	Control n (%)	p value
Skeptic to new food	9 (20)	8 (29)	.372	9 (18)	5 (20)	.804	15 (32)	9 (43)	.383

^a^Chi square

### Discussion

Nutrition the first year of life is important for present and later health. Dietary interventions at this age have therefore been called for [12]. The present intervention aimed to improve child diet by giving maternal guidance on how to make nutritious food for their infant and inform of how to reduce food skepticism. We have previously shown that this intervention improved intake of home-made porridge, reduced intake of industrially produced dinners, and that the intervention in total improved HDL-levels at age 24 months [11].

However, exploring further details, as done in this study, we found no improvement regarding fruits and vegetables, sugary drinks and water consumption. To our knowledge, no previous cooking intervention study in parents of infants has reported on the effects on child diet. Among adults, however, a systematic review reports improved diet, also fruit and vegetable intake, after cooking course participation [13].

The intervention also focused on how to introduce a varied diet, so that the infant would be confident with different flavors from early age to reduce level of food neophobia or skepticism. The results presented show no difference between the intervention and control group regarding food skepticism. We do not know of any other studies that have reported results from dietary and cooking interventions with aim to reduce food skepticism.

There may be several explanations for the null findings regarding fruit and vegetables and beverages. The intervention content and information may have had a too large focus on porridge and industry produced dinners, where differences between the intervention and control group were originally found, and less information on the importance of fruits and vegetables and healthy beverages. Further, there might be other determinants than cooking skills which are important for intake of fruits and vegetables and drinks, as socioeconomic position and accessibility of such food [14].

The null finding regarding food skepticism could also be explained by the diverse focus of the intervention. Regarding food skepticism, the participants were told of the importance of introducing a variety of food to the child [11]. However, other concepts might be more relevant, as focusing on repeated exposure reduce food skepticism [15], and also, introducing taste education lessons, as shown to be effective in school children [16]. In addition, it could be that changing a strong trait as food skepticism [17], may be difficult to do with a 2-day course.

### Limitations

In general, there were few participants in our study, making the results more vulnerable to individual differences. One may presume that the control group were originally very health-conscious and therefore making it difficult to identify effect of the intervention. Assessing vegetable consumption at baseline was difficult and is not included in the presentation since most intake originated from industrialized produced food. A more detailed questionnaire regarding such food should have been used to calculate intake, and is therefore a limitation. We only used one question regarding food skepticism, while most studies use the child food neophobian scale [18], which could have been a better measure to use.

In conclusion, an intervention to improve food variety and reduce food skepticism did not affect intake of fruits and vegetables and intake of water and sugary drinks. Neither did it affect food skepticism among participants. The study is limited by the low number of participants.

## Additional file

**Additional file 1.** Questionnaire.

## Abbreviations

HDL: high density lipoprotein
NCD: non-communicable disease
WHO: World Health Organization
RCT: randomized controlled trial
